# Non-Vitamin K Oral Anticoagulants (NOAC) versus Vitamin K Antagonists (VKA) for Atrial Fibrillation with Elective or Urgent Percutaneous Coronary Intervention: A Meta-Analysis with a Particular Focus on Combination Type

**DOI:** 10.3390/jcm9041120

**Published:** 2020-04-14

**Authors:** Ceren Eyileten, Marek Postula, Daniel Jakubik, Aurel Toma, Dagmara Mirowska-Guzel, Giuseppe Patti, Giulia Renda, Jolanta M. Siller-Matula

**Affiliations:** 1 Department of Experimental and Clinical Pharmacology, Centre for Preclinical Research and Technology (CEPT), Medical University of Warsaw, 02097 Warsaw, Poland; cereneyileten@gmail.com (C.E.); mpostula@wum.edu.pl (M.P.); djakubik@wum.edu.pl (D.J.); dmirowska@wum.edu.pl (D.M.-G.); 2 Department of Internal Medicine II, Division of Cardiology, Medical University of Vienna, Vienna 1090, Austria; aurel.toma@meduniwien.ac.at; 3 Chair of Cardiology, University of Eastern Piedmont and Maggiore della Carità Hospital, 28100 Novara, Italy; giuseppe.patti@uniupo.it; 4 Institute of Cardiology, Department of Neuroscience, Imaging and Clinical Sciences, and Center for Advanced Studies and Technology, CASTG, G. d’Annunzio University, 66100 Chieti-Pescara, Italy; giulia.renda@unich.it

**Keywords:** non-vitamin K oral anticoagulants, vitamin K antagonists, NOAC, VKA, major adverse cardiovascular event, myocardial infarction, stroke, major bleeding, atrial fibrillation, acute coronary syndrome

## Abstract

Background: Our study aims to perform a meta-analysis of benefits and risks associated with the use of non-vitamin K oral anticoagulants (NOAC) versus vitamin K antagonists (VKA) in patients with a percutaneous coronary intervention (PCI) with a particular focus on the combination type: dual vs. dual antithrombotic therapy (DAT: NOAC + single antiplatelet therapy (SAPT) vs. DAT: VKA + SAPT), dual vs. triple antithrombotic therapy (DAT: NOAC + SAPT vs. TAT: VKA + dual antiplatelet therapy (DAPT)) or triple vs. triple antithrombotic therapy (TAT: NOAC+DAPT vs. TAT: VKA+DAPT). Methods: PubMed, EMBASE, and Cochrane databases were searched to identify randomized controlled trials comparing antithrombotic regimens. Four randomized studies (n = 10.969; PIONEER AF-PCI, RE-DUAL PCI, AUGUSTUS, and ENTRUST-AF PCI) were included. The primary outcome was the composite of major bleeding defined by the International Society on Thrombosis and Hemostasis (ISTH) and clinically relevant bleeding requiring medical intervention (CRNM). Secondary outcomes included all-cause mortality, major adverse cardiovascular events (MACE), myocardial infarction (MI), stroke, and stent thrombosis (ST). Results: Combination strategies with NOACs were associated with reduced risk of major bleeding events across different combination strategies as compared to VKA, with the most significant risk reduction when DAT was compared with TAT, namely DAT with NOAC + SAPT was associated with a 37% relative risk reduction (RRR) of major bleeding events as compared to TAT with VKA + DAPT (RR 0.63; 95% CI, 0.50–0.80). The reduction of major bleeding risks is a class effect of NOACs. Combination strategies of NOACs vs. VKAs resulted in a comparable risk of MACE, MI, stroke, ST, or death. Conclusions: Antithrombotic combinations of NOACs (as DAT or TAT) are safer than VKAs with respect to bleeding risk and result in a satisfactory efficacy with no increase of ischemic or thrombotic events in patients undergoing PCI.

## 1. Introduction

Atrial fibrillation (AF) is a global health problem and a common arrhythmia that increases the risk of thromboembolic complications, including stroke and other cardiovascular events [1]. Approximately 20% of the patients with AF need percutaneous coronary intervention (PCI) for concomitant coronary artery disease (CAD) [2,3]. Currently, vitamin K antagonists (VKA) and non–vitamin K antagonist oral anticoagulants (NOAC) are used to prevent AF-related ischemic stroke. For PCI or acute coronary syndrome (ACS), dual antiplatelet therapy (DAPT) with aspirin in addition to a P2Y12 receptor inhibitor (clopidogrel, prasugrel, or ticagrelor) is the gold standard for secondary prevention of ischemic events as myocardial infarction (MI) or stent thrombosis (ST) [4]. In order to reduce the risk of both stroke and coronary ischemic events in this vulnerable population of patients with AF who concomitantly received coronary stents, triple antithrombotic therapies (TAT i.e., a combination of oral anticoagulants (OAC) and DAPT) have been used for the last decade [5]. As a consequence, increased bleeding risks were reported in the literature for such a combination [6,7], with however unknown risk to benefit ratio, especially with the use of NOACs instead of warfarin [8].

Recently, randomized controlled trials (RCTs) have been conducted to compare the safety and efficacy of NOACs versus VKA as a combination with single or dual antiplatelet agents (SAPT/DAPT) in patients with AF undergoing PCI or presenting with ACS [9,10,11,12,13]. Importantly, the ENTRUST trial has been published recently, completing the data on NOAC use in this indication [14]. Unique data on the risks of ST in each treatment arm from the AUGUSTUS trial were published recently [15]. To summarize the existing evidence, we performed a meta-analysis evaluating the safety and efficacy of NOAC vs. VKA with different antiplatelet combination strategies in patients with AF following elective or urgent PCI. A particular focus of this meta-analysis was to perform subgroup analyses focusing on various combinations of antithrombotic strategies as dual or triple antithrombotic therapies (DAT or TAT) while always comparing NOAC vs. VKA.

## 2. Methods

**Figure 1:**
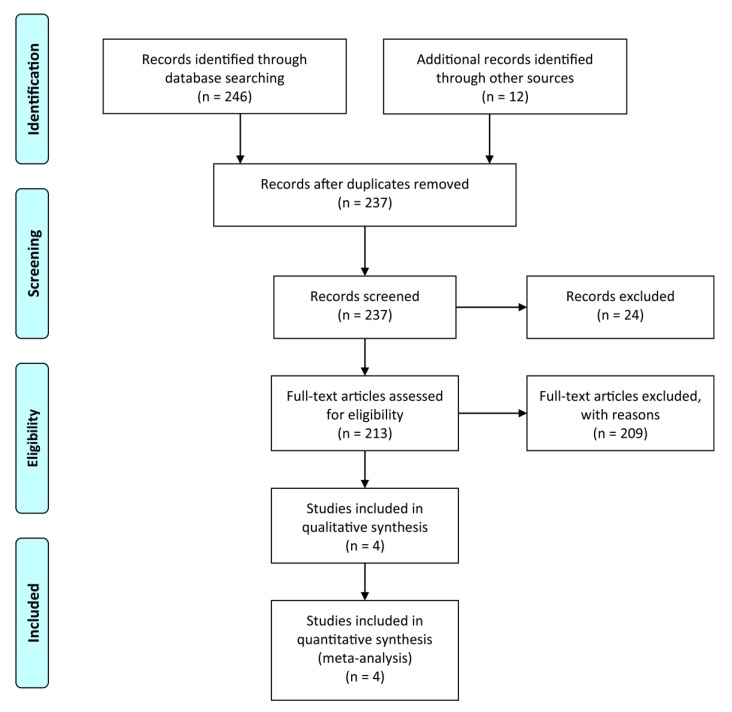
Flow chart of the literature search.

This meta-analysis was performed and reported according to established methods [16]. We searched PubMed and Web of Science using predefined search terms (“percutaneous coronary intervention,” “coronary stenting,” “PCI,” “triple antiplatelet therapy,” “dual antiplatelet therapy,” “triple antithrombotic therapy,” “triple therapy,” “anticoagulants,” “antiplatelets,” “vitamin K antagonists,” “warfarin,” “dabigatran,” “rivaroxaban,” “apixaban,” “edoxaban,” “aspirin,” “clopidogrel,” “prasugrel,” “ticagrelor,” “atrial fibrillation,” and “randomized clinical trial.” Besides, references of prior systematic reviews/meta-analysis, as well as abstracts from major cardiology meetings, were screened for related studies. The search was performed until September 2019. Two reviewers (CE and MP) independently and in duplicate applied the selection criteria. Studies were rejected if one could determine, from the title or abstract or both, that the study was not suitable for inclusion. The full text of the study was obtained and evaluated if an article could not be excluded with certainty. Excluded studies were compared, and any disagreement was resolved through discussion between the reviewers. We also checked the references of manually retrieved studies for additional trials. We used no language restrictions. Only full-text articles were included, Figure 1. Eligible reports were assessed for methodological quality (Supplemental Figure S1). Selected studies had to be RCTs comparing NOAC with SAPT or DAPT vs. VKA with SAPT or DAPT in patients with AF following PCI or ACS. The primary exclusion criteria were observational non-randomized studies, registry data, retrospective analysis, ongoing trials without results, editorials, case series, and duplicate studies.

The primary safety outcome was the composite of major bleeding defined by the International Society on Thrombosis and Hemostasis (ISTH) and clinically relevant bleeding requiring medical intervention (CRNM). The primary efficacy outcome was “trial-defined major adverse cardiovascular events (MACE),” which followed the definition of MACE in the respective trials (Supplementary Table S1). Secondary efficacy outcomes were the individual components i.e., the occurrence of all-cause death, MI, stroke, or ST (definite or probable).

### Statistics

Variables are reported as numbers or percentages as appropriate. Risk ratios (RR) were computed from individual studies and pooled according to the inverse variance weighted model with 95% confidence intervals (CIs) using RevMan (Version 5.3. Copenhagen: The Nordic Cochrane Centre, The Cochrane Collaboration, 2014) as appropriate [17]. We assessed the studies for clinical and statistical heterogeneity. To assess the statistical heterogeneity, we calculated the *I*^2^ index and a *p*-value. Percentages lower than 25% (*I*^2^ = 25), 50% (*I*^2^ = 50), and 75% (*I*^2^ = 75) correlate to low, medium, and high heterogeneity, respectively [18]. The methodological quality of the randomized trials was assessed by Cochrane’s Collaboration tool for assessing the risk of bias. For each trial, bias was assessed qualitatively as low risk, intermediate risk, or high risk of bias by independent investigators. Publication bias was not assessed as there were a small number of studies (<5) included in the analysis. A two-tailed *p*-value of <0.05 was considered significant. The number needed to prevent major bleedings and the number needed to cause harm by increasing the risk of stent thrombosis were calculated according to the established statistical methods.

We performed a sensitivity analysis by including subgroups for the type of comparison (dual vs. dual therapy: DAT vs. DAT; dual vs. triple therapy: DAT vs. TAT; triple vs. triple therapy: TAT vs. TAT), and the type of NOAC (dabigatran vs. rivaroxaban vs. apixaban vs. edoxaban).

## 3. Results

A total of 237 studies were screened for eligibility, out of which 4 Phase 3–4 RCTs assessing the strategy of NOAC with either a P2Y12 inhibitor or DAPT vs. VKA (primarily represented by warfarin) with a P2Y12 inhibitor or DAPT in AF patients following PCI were included in the final analysis (Supplementary Figure S1). All four studies were conducted between 2013 and 2019, comprising 10,969 patients with atrial fibrillation, of whom 55.54% had ACS, and about a quarter was female. For the analysis of the risk of ST from the AUGUSTUS trial, recently published data in patients receiving coronary stent at index hospitalization were used [15].

**Figure 2:**
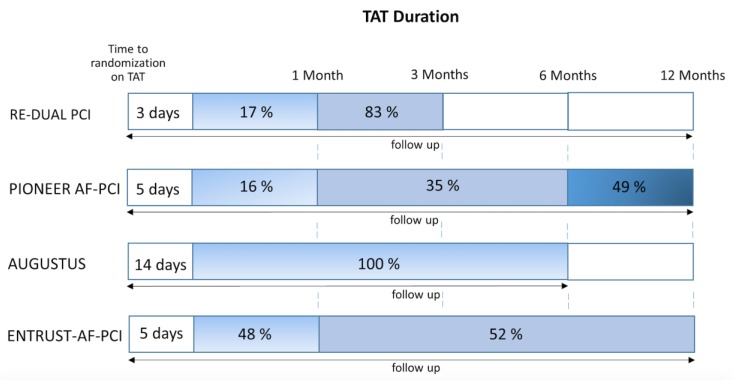
Heterogeneity of treatment algorithms of trials comparing non–vitamin K antagonist oral anticoagulants (NOACs) vs. vitamin K antagonists (VKAs), illustrating (I) the time on triple antithrombotic therapy (TAT) until randomization, (II) the percentage of patients on TAT treatment with respect to time (in blue) if randomized to the TAT group, and (III) the follow-up duration (arrow).

Follow-up ranged from 6 to 14 months. Of the P2Y12 inhibitors, clopidogrel was the most commonly used (92%), while ticagrelor and prasugrel were used in 7% and 1% cases, respectively. Details of the design of the included studies are listed in Table 1 and Table 2 and Supplementary Table S1. The particular time to randomization, duration of TAT, and follow-up time are shown in Figure 2. All trials were judged to be at low risk of bias via the Cochran’s Collaboration tool for risk assessment (Supplementary Figures S1 and S2).

### 3.1. Dual Antithrombotic Therapy (DAT) Consisting of a NOAC and a Single Antiplatelet Drug (SAPT: P2Y12 Inhibitor) vs. Triple Antithrombotic Therapy (TAT) Consisting of a VKA and Dual Antiplatelet Therapy (DAPT: P2Y12 Inhibitor and Aspirin)

**Figure 3:**
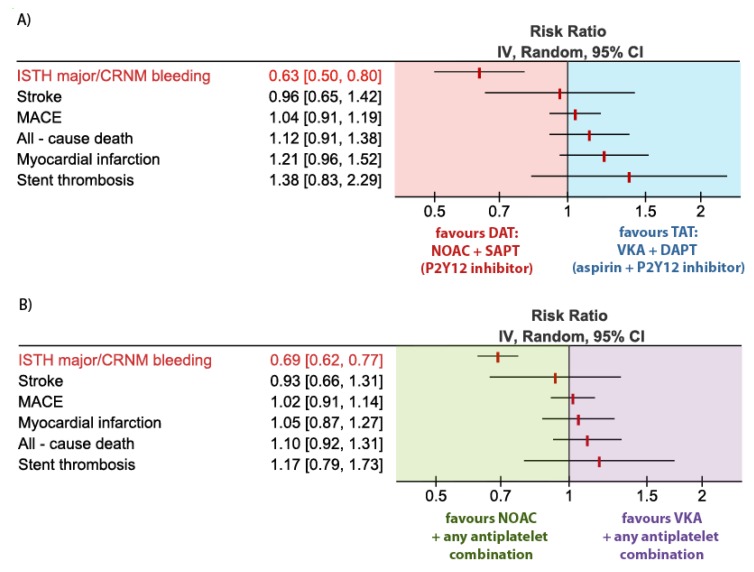
Forest plot for the main outcome parameters according to the treatment groups: (**A**) Dual antithrombotic therapy (DAT) consisting of non–vitamin K antagonist oral anticoagulants (NOAC) + single antiplatelet therapy (SAPT) vs. triple antithrombotic therapy (TAT) consisting of vitamin K antagonist (VKA) + dual antiplatelet therapy (DAPT); (**B**) NOAC (+ any antiplatelet combination: SAPT or DAPT) vs. VKA (+ any antiplatelet combination: SAPT or DAPT).

ISTH major or CRNM bleeding events occurred in 14.6% in the DAT (NOAC + SAPT) group vs. 23.1% in the TAT (VKA + DAPT) group. A combination strategy of DAT (NOAC + SAPT) was therefore associated with a 37% relative risk reduction (RRR) in ISTH major or CRNM bleeding events as compared to TAT. VKA + DAPT; RR 0.63; 95% CI, 0.50–0.80; *p =* 0.001; *I*^2^ = 86% Figure 3A. By using NOAC + SAPT instead of VKA + DAPT a number needed to prevent one major bleeding would be 12. This translates into 84 fewer bleeding events per 1 thousand patients when treated with NOAC + SAPT instead of VKA + DAPT.

MACE occurred in 9.0% in the DAT (NOAC + SAPT) group vs. 8.7% in the TAT (VKA + DAPT) group, resulting in no statistical difference in the risk of MACE between the groups. RR 1.04; 95% CI, 0.91–1.19; *p =* 0.57; *I*^2^ = 0%, Figure 3A.

Myocardial infarction occurred in 3.6% in the DAT (NOAC + SAPT) group vs. 3.0% in the TAT (VKA + DAPT) group, resulting in no statistical difference in the risk of myocardial infarction between the groups. RR 1.21; 95% CI, 0.96–1.52; *p =* 0.11; *I*^2^ = 0%, Figure 3A.

Stroke occurred in 1.1% in the DAT (NOAC + SAPT) group vs. 1.2% in the TAT (VKA + DAPT) group, resulting in no statistical difference in the risk of stroke between the groups. RR 0.96; 95% CI, 0.65–1.42; *p =* 0.84; *I*^2^ = 6%, Figure 3A.

All-cause death occurred in 3.9% in the DAT (NOAC + SAPT) group vs. 3.5% in the TAT (VKA + DAPT) group, resulting in no statistical difference in the risk of stroke between the groups. RR 1.12; 95% CI, 0.91–1.38; *p =* 0.29; *I*^2^ = 0%, Figure 3A.

Stent thrombosis (ST) occurred in 1.3% in the DAT (NOAC + SAPT) group vs. 0.9% in the TAT (VKA + DAPT) group, resulting in no statistical difference in the risk of ST between the groups. RR 1.38; 95% CI, 0.87–2.20; *p =* 0.17; *I*^2^ = 0%, Figure 3A.

### 3.2. NOAC with Any Antiplatelet Combination vs. VKA with Any Antiplatelet Combination

ISTH major or CRNM bleeding events occurred in 14.6% in the NOAC group vs. 21.1% in the VKA group. NOAC use was associated with a 31% RRR in ISTH major or CRNM bleeding events as compared to VKA. RR 0.69; 95% CI, 0.62–0.77; *p* < 0.001; *I*^2^ = 56%, Figure 3B. By using NOAC instead of VKA as a part of antithrombotic combination therapy, a number needed to prevent one major bleeding is 15. This translates into 67 fewer bleeding events per 1 thousand patients treated with NOAC instead of VKA.

MACE occurred in 8.4% in the NOAC group vs. 8.2% in the VKA group, resulting in no statistical difference in the risk of MACE between the groups. RR 1.02; 95% CI, 0.91–1.14; *p =* 0.73; *I*^2^ = 0%, Figure 3B.

Myocardial infarction occurred in 3.3% in the NOAC group vs. 3.2% in the VKA group, resulting in no statistical difference in the risk of myocardial infarction between the groups. RR 1.05; 95% CI, 0.87–1.27; *p =* 0.61; *I*^2^ = 0%, Figure 3B.

Stroke occurred in 1.1% in the NOAC group vs. 1.2% in the VKA group, resulting in no statistical difference in the risk of stroke between the groups. RR 0.93; 95% CI, 0.66–1.31; *p =* 0.68; *I*^2^ = 6%, Figure 3B.

All-cause death occurred in 3.6% in the NOAC group vs. 3.3% in the VKA group, resulting in no statistical difference in the risk of stroke between the groups. RR 1.10; 95% CI, 0.92–1.31; *p =* 0.30; *I*^2^ = 0%, Figure 3B.

ST occurred in 0.95% in the NOAC group vs. 0.82% in the VKA group, resulting in no statistical difference in the risk of ST between the groups. RR 1.17; 95% CI, 0.79–1.74; *p =* 0.44; *I*^2^ = 0%, Figure 3B.

### 3.3. Subgroup Analyses

#### 3.3.1. ISTH Major or CRNM Bleeding Events in Patients Treated with Combinations Consisting of a NOAC vs. a VKA

**Figure 4:**
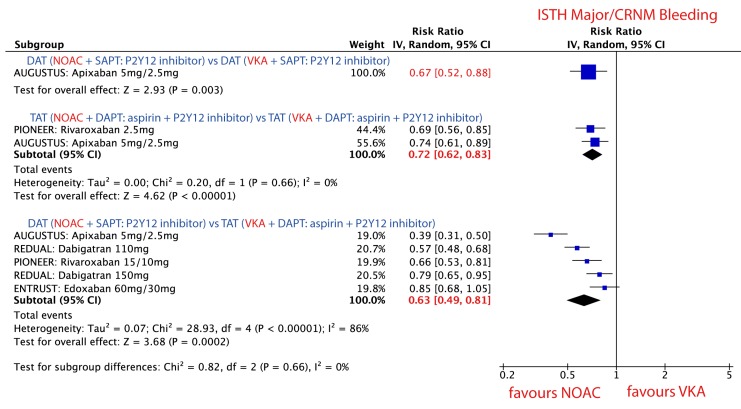
Forest plot for the main safety outcome parameter ISTH major/CRNM bleeding in patients treated with novel oral anticoagulant (NOAC) vs. vitamin K antagonist (VKA) according to three different combination strategies. Dual antithrombotic therapy (DAT); single antiplatelet therapy (SAPT); triple antithrombotic therapy (TAT); dual antiplatelet therapy (DAPT).

There was no difference in the direction of the effect of NOACs vs. VKA on the risk of bleeding events, but the magnitude of the effect varied for different combination strategies, Figure 4.

#### 3.3.2. DAT vs. DAT: NOAC + P2Y12 Inhibitor vs. VKA + P2Y12 Inhibitor

Use of *NOAC + P2Y12* inhibitor was associated with a 33% RRR in ISTH major or CRNM bleeding events as compared to *VKA + P2Y12* inhibitor. RR 0.67; 95% CI, 0.52–0.88; *p =* 0.003, Figure 4.

#### 3.3.3. TAT vs. TAT: NOAC + P2Y12 Inhibitor + Aspirin vs. VKA + P2Y12 Inhibitor + Aspirin

Use of *NOAC + P2Y12* inhibitor + aspirin was associated with a 28% RRR in ISTH major or CRNM bleeding events as compared to *VKA + P2Y12* inhibitor + aspirin. RR 0.72; 95% CI, 0.62–0.83; *p* < 0.001; *I*^2^ = 0%, Figure 4.

#### 3.3.4. DAT vs. TAT: NOAC + P2Y12 Inhibitor vs. VKA + P2Y12 Inhibitor + Aspirin

Use of *NOAC + P2Y12* inhibitor was associated with 37% RRR in ISTH major and CRNM bleeding events as compared to *VKA + P2Y12* inhibitor + aspirin. RR 0.63; 95% CI, 0.49–0.81; *p* < 0.001; *I*^2^ = 0% Figure 4.

#### 3.3.5. Stent Thrombosis (ST) in Patients Treated with Combinations Consisting of NOAC vs. VKA

**Figure 5:**
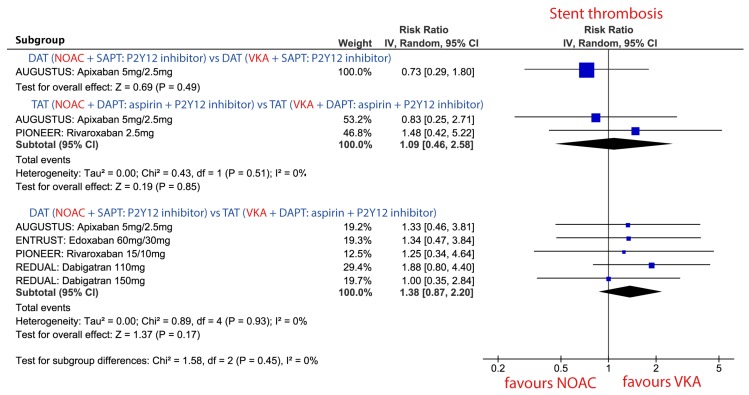
Forest plot for the risk of stent thrombosis (ST: definite or probable) in patients treated with novel oral anticoagulant (NOAC) vs. vitamin K antagonist (VKA) according to three different combination strategies. Dual antithrombotic therapy (DAT); single antiplatelet therapy (SAPT); triple antithrombotic therapy (TAT); dual antiplatelet therapy (DAPT).

The risk of ST did not statistically differ between NOAC vs. VKA in none of the three different combination strategies. Nevertheless, there was a trend toward a higher risk of ST when DAT with NOAC was compared against TAT with VKA Figure 5.

#### 3.3.6. Major Adverse Cardiac Events (MACE) in Patients Treated with Combinations Consisting of NOAC vs. VKA

The risk of MACE did not statistically differ between NOAC vs. VKA in none of the three different combination strategies (Supplementary Figure S3).

#### 3.3.7. All-Cause Mortality in Patients Treated with Combinations Consisting of NOAC vs. VKA

The risk of death did not statistically differ between NOAC vs. VKA in none of the three different combination strategies (Supplementary Figure S4).

### 3.4. Sensitivity Analysis

#### 3.4.1. ISTH Major/CRNM Bleeding

The direction and the magnitude of the effect did not change after the exclusion of single studies, Table 3. The magnitude of the effect was strongest when apixaban + SAPT (DAT) was compared with VKA + DAPT (TAT) in the AUGUSTUS trial, Table 3.

#### 3.4.2. Stent Thrombosis

The direction and the magnitude of the effect did not change after the exclusion of single studies (Table 3). The strongest trend toward ST was found in the REDUAL trial, with 110 mg dabigatran (Table 3).

## 4. Discussion

Our meta-analysis in over ten thousand patients confirms that NOACs as part of diverse antithrombotic combinations are safer than VKAs with respect to major bleeding events. The magnitude of the effect was larger when NOACs were used as part of a DAT than TAT; NOACs as part of DAT were associated with a 37% RRR of major bleeding events as compared to VKA as a part of TAT. The RRR was 31% when TAT with NOAC was compared with TAT with VKA. The homogeneity in the effect of NOACs underscores their role in improving patients’ safety in the setting of AF and PCI.

Of special interest, a combination of NOAC with SAPT/DAPT did not increase the risk of MACE, MI, stroke, ST, or death as compared to VKA with SAPT/DAPT in patients undergoing PCI. The neutral effect on mortality of NOAC + SAPT in comparison to VKA + DAPT might be a consequence of reduced mortality due to lower bleeding events. This is a very important finding as single studies were underpowered to make conclusions about the efficacy issues.

The optimal antithrombotic strategy and especially its duration for patients with AF undergoing PCI or presenting with an ACS is still a matter of debate. DAT or TAT aims at effectively preventing thromboembolic events with an acceptable risk of bleeding complications. The pivotal clinical trials on antithrombotic therapy were designed to test efficacy in preventing thromboembolic events rather than reduction of bleeding [19,20,21]. Importantly, recently conducted RCTs comparing the combination of NOACs with antiplatelet drugs versus VKA with antiplatelet drugs in patients with AF and PCI/ACS primarily assessed bleeding events [9,11,12,13,14]. Of interest, none of these contemporary trials of NOACs were designed to be large enough to detect small but potentially meaningful differences in the incidence of ischemic events.

According to the latest consensus document on the management of antithrombotic therapy in AF patients presenting with ACS and/or undergoing PCI, TAT should be only used in patients at low bleeding risk and a very short duration (one week) of such a combination should be preferred in those at high bleeding risk [22]. An issue of special importance to discuss is the difference in the duration of TAT in the VKA group in each study included in our meta-analyses. In the PIONEER-AF PCI, 49% of the patients were assigned to 1-year treatment with standard TAT, while 16% and 35% of patients were to be treated for 1 and 6 months, respectively. However, only 22% of the patients receiving triple therapy for 1 year completed treatment on the scheduled termination date [11,13]. In contrast, in the RE-DUAL PCI trial, TAT was used for 1 month in patients who received a bare-metal stent (17%) and for 3 months in patients who received a drug-eluting stent (83%) [12]. In contrast to current international guidelines and abovementioned studies, which recommend TAT for a maximum of 6 months only in AF patients at very high risk of ischemic events after PCI, all patients in the AUGUSTUS trial received TAT for the whole period of 6 months [10]. Based on the ENTRUST-AF PCI trial design, 48% of patients (stable CAD) were on TAT for one month, and patients with ACS (52%) received TAT for 6 to 12 months [14]. Therefore, the included trials varied in the duration of antithrombotic combinations, which underlines the heterogeneity of the study designs, Figure 2. Nevertheless, after the trials were pooled into a meta-analysis, a homogenous effect was found for the majority of outcomes.

Several fundamental questions surrounding the risk of ST while treating patients with a NOAC and P2Y12 inhibitor and omitting aspirin also during the first weeks after stent implantation remain a matter of debate. Our meta-analysis shows that when aspirin is omitted and only DAT with NOAC+SAPT is used early after elective PCI or ACS with PCI (for 1 week on average Figure 2), the risk of ST is not statistically higher as compared to TAT with VKA+DAPT. This finding is in contrast to two previous meta-analyses [14,23], indicating a borderline significance toward increased ST risk while on NOAC. This difference in the results might be due to two major issues. First, only recently, the detailed data on ST rates for each arm from the AUGUSTUS study were published [15]. Therefore, the previous two meta-analyses included different rates of ST (definite and probable) in the DAT group with apixaban (*n* = 21 vs. *n* = 8 in our meta-analysis), which most probably was based on authors personal communication, as the data were not available. Additionally, the meta-analysis by Gargiulo et al. [23], which was published later, stated that the ST rate was derived not from the original study but from the first meta-analysis by Vrancx et al. [14]. Therefore, it cannot be excluded that some inconsistency was repeated. Of special interest is the fact that the *n* = 21 of ST was used in both meta-analyses for the DAT group, irrespectively, whether apixaban was used with P2Y12 inhibitor or apixaban/warfarin with P2Y12 inhibitor. Another major issue is the fact that one-third of the patients in the AUGUSTUS study did not receive a stent at the index event, and therefore were not at risk for ST. In the two previous meta-analyses, the ST data from the overall cohort was used. In contrast, we used the ST data from the AUGUSTUS population receiving a coronary stent, which might result in more robust but diverging results.

In the AUGUSTUS trial patient population with AF and recent stenting, stent thrombosis was rare (<1% over 6 months), occurred early after PCI, and was numerically less frequent in patients receiving apixaban than VKA [15]. A trend toward an elevated risk of ST was observed in the RE-DUAL PCI trial, in the DAT group randomized to 110 mg of dabigatran with P2Y12 inhibitor [12]. If robust, postulated increased risk of ST with use of NOAC+SAPT in the previous meta-analyses would be of particular clinical importance as the question of an optimal time period to drop aspirin after PCI in AF patients remains unanswered yet [23]. As patients have the highest risk for coronary ischemic events in the first days-weeks after PCI, patients in the recent 4 RCTs were on triple therapies for some intervals from the index event to enrollment (PIONEER AF-PCI ≤ 3 days, RE-DUAL PCI ≤ 5 days, AUGUSTUS ≤ 14 days, and ENTRUST-AF PCI ≤ 5 days; Figure 2) [10,11,12,14]. Despite the 3–14 days of TAT in these trials, Figure 3, some risk of stent thrombosis exists. Therefore, a short time period for a triple therapy (at least one month), might still be recommended if the bleeding risk is low.

Another critical question that needs to be answered is the optimal combination of NOAC with P2Y12 inhibitors i.e., clopidogrel, ticagrelor, or prasugrel in terms of safety, but also in terms of risk of thrombotic events [24]. Clopidogrel is still the most widely used P2Y12 inhibitor in patients requiring both NOAC and DAPT/SAPT. Nevertheless, a considerable number of clopidogrel-treated patients experience adverse thrombotic events in whom insufficient P2Y12-inhibition by clopidogrel and a consequential high on-treatment platelet reactivity is a common finding [25,26]. Therefore, it might be that patients treated with a NOAC and clopidogrel would have an increased risk of stent thrombosis because of an insufficient effect of clopidogrel. The next logical question would be whether a combination of a NOAC and aspirin would achieve better efficacy than NOAC with clopidogrel.

Furthermore, variability in ADP-induced platelet aggregation under treatment with clopidogrel can be overcome with prasugrel and ticagrelor [3]. It should be noted that in the RE-DUAL PCI trial only 12% of patients were taking ticagrelor, whereas, in the PIONEER AF-PCI, AUGUSTUS, and ENTRUST- AF PCI trials, the numbers were even lower with 5%, 6%, and 7% of patients on ticagrelor, respectively, and a minority of patients were taking prasugrel (1% in AUGUSTUS, PIONEER AF-PCI and ENTRUST- AF PCI, and 0% in RE-DUAL PCI trial). The sub-analysis of RE-DUAL PCI trial showed that the major or clinically relevant non-major bleeding events according to ISTH occurred significantly higher in the ticagrelor vs. clopidogrel groups: 26% vs. 20% (adjusted hazard ratio-HR 1.35, 95% CI 1.05–1.72) [27]. In line, the rates of death and thromboembolic events with ticagrelor were also higher as compared to clopidogrel (18.7% vs. 12.9%; HR 1.34, 95% CI 1.00–1.82) [27]. This result indicates that potent P2Y12 inhibitors might not be a drug of the first choice in antithrombotic combination therapies [22]. In the population of AF patients undergoing stent implantation with a high risk of thrombotic events more individualized approach might be considered with alternative strategies like a combination of NOAC with aspirin, however, has to be investigated in future trials [28].

Another critical issue that should be discussed here is the significant differentiation of the duration of TAT between stable CAD and ACS. The guidelines and consensus documents recommend clearly short TAT for stable CAD and longer TAT for ACS. In the PIONEER-AF PCI, RE-DUAL PCI, and ENTRUST-AF PCI, around 50% participants were presenting with ACS, in the AUGUSTUS trial this proportion was highest (60%). Moreover, in the AGUSTUS trial, almost 24% of patients had medically managed ACS with no clear indication for TAT. Moreover, according to the latest update, TAT with NOAC is recommended only for one month after elective PCI in patients with high thromboembolic risk and low risk of bleeding and for 3–6 months after ACS [22]. In the majority of included studies, however, TAT was used for a much longer duration 3–12 months also in elective PCI.

Another interesting point that could potentially influence the risk of bleeding is the dose adjustment of a drug in patients with renal insufficiency. Rivaroxaban was reduced from 15 mg to 10 mg once daily for patients with a creatinine clearance of 30 to 50 mL per minute in the PIONEER-AF trial. It is noteworthy that this study tested two lower rivaroxaban doses (15 mg and 2.5 mg) as an alternative to approved drug regimen in AF patients (Rivaroxaban 20 mg when combined with P2Y12 inhibitor). Edoxaban was reduced to 30 mg instead of 60 mg once daily if moderate or severe renal impairment was observed (calculated CrCl 15–50 mL/min) in the ENTRUST trial [11,14]. In the AUGUSTUS trial, patients were directed to take 2.5 mg twice daily apixaban if they met two or more of the following dose-reduction criteria: were at least 80 years of age, had a weight of no more than 60 kg, or had a creatinine level of at least 1.5 mg per deciliter (133 μmol per liter) [10].

A novelty in our meta-analysis is the inclusion of all NOAC types as a part of antithrombotic therapies for AF with ACS/PCI and the unique inclusion of AUGUSTUS population receiving stent implantation. Previously published meta-analyses included studies comparing DAT vs. TAT irrespective of the oral anticoagulant type in the comparator arm [5,29]. Currently, after the results of ENTRUST-AF PCI were published, we included only studies with NOAC as a part of antithrombotic therapy as compared to warfarin-based TAT. As it was previously mentioned, from the PIONEER-AF PCI study, we also included a subgroup of patients receiving 2.5 mg of rivaroxaban as a part of TAT, even though such a dose is not recommended for stroke prevention.

There are some limitations of this study. Most importantly, different durations of TAT use and of follow-up, as well as differences in patients’ characteristics between the trials underline the heterogonous nature of included trials. Nevertheless, the sensitivity analyses underscored the robustness of the main finding.

## 5. Conclusions

This meta-analysis shows that NOACs as a part of DAT or TAT is safer than VKA with respect to bleeding risk with a comparable risk for major adverse ischemic events in patients undergoing PCI. Nevertheless, the optimal antithrombotic regimen with a NOAC should be cautiously selected based on a number of considerations like acute vs. elective procedure, individual bleeding risk, and thromboembolic risk.

## 6. Summary Box

### 6.1. What Is Already Known on This Topic

Currently, vitamin K antagonists (VKA) and non–vitamin K antagonist oral anticoagulants (NOAC) are used to prevent ischemic stroke related to atrial fibrillation (AF).

Recently, randomized controlled trials (RCTs) have been conducted to compare the safety and efficacy of NOACs versus VKA as a combination with single or dual antiplatelet agents (SAPT/DAPT).

The evidence regarding benefits and risks associated with the use of all available NOACs (i.e., rivaroxaban, edoxaban, apixaban, and dabigatran) versus VKA with single (SAPT) or dual antiplatelet therapy (DAPT) in AF patients following elective percutaneous coronary intervention (PCI) or acute coronary syndrome (ACS) with stenting has not been fully investigated yet.

### 6.2. What This Study Adds

Antithrombotic combinations of NOACs (as part of TAT or DAT) are safe and reduce the risk of bleeding events in a significant and relevant magnitude as compared to VKA (as part of DAT or TAT).

The efficacy of antithrombotic combinations of NOACs (as part of TAT or DAT) is satisfactory and results in a comparable risk of major ischemic events as VKA (as part of DAT or TAT).

## Figures and Tables

**Table 1:** Baseline characteristics of the patients *.

	PIONEER AF-PCI			RE-DUAL PCI			ENTRUST AF PCI		AUGUSTUS			
	Rivaroxaban 15 mg QD + P2Y_12_ Inhibitor (*N* = 709)	Rivaroxaban 2.5 mg + DAPT (*N* = 709)	VKA + DAPT (*N* = 706)	Dabigatran 110 mg + P2Y_12_ Inhibitor (*N* = 981)	Dabigatran 150 mg + P2Y_12_ Inhibitor (*N* = 763)	VKA + DAPT (*N* = 981)	Edoxaban + P2Y_12_ Inhibitor (*N* = 751)	VKA + DAPT (*N* = 755)	Apixaban + P2Y_12_ Inhibitor (*N* = 2306)	VKA + P2Y_12_ Inhibitor (*N* = 2308)	Aspirin (*N* = 2307)	Aspirin-Matched Placebo (*N* = 2307)
Age (year)	70.4 ± 9.1	70.0 ± 9.1	69.9 ± 8.7	71.5 ± 8.9	68.6 ± 7.7	71.7 ± 8.9	69 (63–77)	70 (64–77)	70.4 (64.1–77.2)	70.9 (64.3–77.2)	70.8 (64.4–77.3)	70.6 (63.8–77.2)
Female (%)	25.5	24.5	26.6	25.8	22.4	23.5	26	25	29.1	28.9	30.2	27.8
BMI, median (IQR)	28.6 (25.7–32.4)	28.4 (25.6–32.1)	29.0 (25.8–32.8)	NR	NR	NR	NR	NR	NR	NR	NR	NR
Diabetes mellitus (%)	28.8	28.1	31.3	36.9	34.1	37.9	34	34	36.5	36.2	36.5	36.2
Hypertension (%)	73.3	73.2	75.4	NR	NR	NR	90	91	88.6	88.0	88.0	88.5
Dyslipidemia (%)	42.6	41.6	44.8	NR	NR	NR	66	64	NR	NR	NR	NR
Current smoker (%)	5.2	7.9	6.8	NR	NR	NR	NR	NR	NR	NR	NR	NR
History OF MI (%)	19.8	25.4	25.4	24.2	25.4	27.3	25	23	NR	NR	NR	NR
Heart Failure (%)	25.4	26.4	24.8	NR	NR	NR	56	54	42.4	43.2	42.6	43.0
History of CABG (%)	NR	NR	NR	9.9	10.4	11.3	6	6	NR	NR	NR	NR
History of PCI (%)	NR	NR	NR	33.2	31.3	35.4	26	26	NR	NR	NR	NR
Type of index event (%)												
ACS	51.5	53.2	52.2	51.9	51.2	48.4	52	52	61.8	60.5	60.7	61.7
NON-ACS	48.5	46.8	47.8	48.1	48.8	51.6	48	48	38.2	39.5	39.3	38.3
Type of stent (%)												
Drug-eluting	65.4	66.8	66.5	82.1	81.5	84.6	NR	NR	NR	NR	NR	NR
Bare-metal stent	32.6	31.2	31.8	15.1	16.1	13.6	NR	NR	NR	NR	NR	NR
Drug-eluting and bare-metal stent	2.0	2.0	1.7	1.9	1.3	1.2	NR	NR	NR	NR	NR	NR
Type of P2Y12i (%)												
Clopidogrel	93.1	93.7	96.3	86.4	86.9	90.3	93	92	93.4	91.8	92.1	93.2
Ticagrelor	5.2	4.8	3.0	12.6	12.1	7.8	7	8	5.4	7.1	6.5	5.9
Prasugrel	1.7	1.6	0.7	0	0	0	<1	<1	1.2	1.1	1.4	0.9
Type of P2Y12i CLP/TIG/PRS	94%/4%/1%			88%/12%/0%			92,5%/7,5%/<1%		92.6%/6.2%/1.1%			
CHA_2_DS_2_-VASc score ‡	3.7 ± 1.7	3.8 ± 1.6	3.8 ± 1.6	3.7 ± 1.6	3.3 ± 1.5	3.8 ± 1.5	4.0 (3.0-5.0)	4.0 (3.0-5.0)	3.9 ± 1.6	4.0 ± 1.6	3.9 ± 1.6	3.9 ± 1.6
HAS-BLED score †	3.0 ± 0.91	2.92 ± 0.96	2.98 ± 0.92	2.7 ± 0.7	2.8 ± 0.8	2.6 ± 0.7	3.0 (2.0–3.0)	3.0 (2.0–3.0)	2.9 ± 1.0	2.9 ± 0.9	2.8 ± 0.9	2.9 ± 1.0

* Values are reported as mean ± SD. MI, myocardial infarction; CABG, coronary artery bypass grafting; PCI, percutaneous coronary intervention; ACS, acute coronary syndrome; DAPT, dual antiplatelet therapy; VKA, vitamin K antagonist; PIONEER AF, open-label, randomized, controlled, multicenter study exploring two treatment strategies of rivaroxaban and a dose-adjusted oral vitamin K antagonist treatment strategy in subjects with atrial fibrillation; RE-DUAL PCI, randomized evaluation of dual antithrombotic therapy with dabigatran versus triple therapy with warfarin in patients with non-valvular atrial fibrillation undergoing percutaneous coronary intervention; AUGUSTUS, an open-label, 2 × 2 factorial, randomized controlled, clinical trial to evaluate the safety of apixaban vs. vitamin K antagonist and aspirin vs. aspirin placebo in patients with atrial fibrillation and acute coronary syndrome or percutaneous coronary intervention; ENTRUST-AF-PCI, edoxaban treatment versus vitamin K antagonist in patients with atrial fibrillation undergoing percutaneous coronary intervention. ^‡^ The CHA2DS2-VASc score reflects the risk of stroke, with values ranging from 0 to 9 and higher scores indicating greater risk. ^†^ The HAS-BLED score reflects the risk of major bleeding among patients with atrial fibrillation who are receiving anticoagulant therapy, with values ranging from 0 to 9 and with higher scores indicating greater risk. § ACS in PIONEER AF-PCI study is defined as “NSTEMI, STEMI, and unstable angina”; in RE-DUAL PCI as “acute coronary syndrome”; in AUGUSTUS trial as “acute coronary syndrome and PCI and medically managed acute coronary syndrome”; in ENTRUST AF PCI as “acute coronary syndrome.”

**Table 2:** Treatment strategies in trials of dual or triple antithrombotic therapy.

Trial Name	Subgroup	Anticoagulant	Duration (Months)	Patients n/N (%)
PIONEER AF-PCI	DAT	Rivaroxaban 15 mg	12	709/709 (100%)
		Clopidogrel 75 mg	12	
	TAT	Rivaroxaban 2.5 mg or 15 mg	1	109/709 (15.4%)
			6	248/709 (35%)
			12	352/709 (49.6%)
		ASA 75–100 mg	12	709/709 (100%)
		Clopidogrel 75 mg	1	109/709 (15.4%)
			6	248/709 (35%)
			12	352/709 (49.6%)
	TAT	VKA (INR 2.0–3.0)	12	706/706 (100%)
		ASA 75–100 mg	12	
		Clopidogrel 75 mg	1	113/697 (16.2%)
			6	243/697 (34.9%)
			12	341/697 (48.9%)
RE-DUAL PCI	DAT Dabigatran 110 mg	Dabigatran 110 mg	14	981/981 (100%)
		Clopidogrel 75 mg	14	
	DAT Dabigatran 150 mg	Dabigatran 150 mg	14	763/763 (100%)
		Clopidogrel 75 mg	14	
	TAT	VKA (INR 2.0–3.0)	14	981/981 (100%)
		ASA 75–100 mg	1	171/981 (17%)
			3	810/981 (83%)
		Clopidogrel 75 mg	14	981/981 (100%)
ENTRUST AF PCI	DAT	Edoxaban 60 mg	12	751/751 (100%)
		Clopidogrel 75 mg	12	
	TAT	VKA (INR 2.0–3.0)	12	755/755 (100%)
		ASA 100 mg	1 to 12	
		Clopidogrel 75 mg	12	
AUGUSTUS	DAT	Apixaban 5 mg	6	1153/1153 (100%)
		Clopidogrel 75 mg	6	
	TAT	VKA (INR 2.0–3.0)	6	1154/1154 (100%)
		ASA 81 mg	6	
		Clopidogrel 75 mg	6	

PIONEER AF, open-label, randomized, controlled, multicenter study exploring two treatment strategies of rivaroxaban and a dose-adjusted oral vitamin K antagonist treatment strategy in subjects with atrial fibrillation; RE-DUAL PCI, randomized evaluation of dual antithrombotic therapy with dabigatran versus triple therapy with warfarin in patients with non-valvular atrial fibrillation undergoing percutaneous coronary intervention; AUGUSTUS, an open-label, 2 × 2 factorial, randomized controlled, clinical trial to evaluate the safety of apixaban vs. vitamin K antagonist and aspirin vs. aspirin placebo in patients with atrial fibrillation and acute coronary syndrome or percutaneous coronary intervention; ENTRUST-AF-PCI, edoxaban treatment versus vitamin K antagonist in patients with atrial fibrillation undergoing percutaneous coronary intervention.

**Table 3:** Sensitivity analysis regarding the two major endpoints for comparison of DAT (NOAC + SAPT) vs. TAT (VKA + DAPT) by excluding single studies.

ISTH Major/CRNM Bleeding		
**Study**	**RR (95% CI) for Each Study**	**RR (95% CI) for the Total Effect After Exclusion of Each Single Study**
AUGUSTUS: Apixaban 5mg/2.5mg	0.39 [0.31, 0.50]	0.71 [0.59, 0.84]
REDUAL: Dabigatran 110mg	0.57 [0.48, 0.68]	0.65 [0.47, 0.89]
PIONEER: Rivaroxaban 15/10mg	0.66 [0.53, 0.81]	0.62 [0.46, 0.85]
REDUAL: Dabigatran 150mg	0.79 [0.65, 0.95]	0.60 [0.45, 0.80]
ENTRUST: Edoxaban 60mg/30mg	0.85 [0.68, 1.05]	0.59 [0.45, 0.77]
**total**	0.63 [0.49, 0.78]	
**Stent Thrombosis (Definite or Probable)**		
**Study**	**RR (95% CI)**	**RR (95% CI) for the Total Effect After Exclusion of Each Single Study**
AUGUSTUS: Apixaban 5mg/2.5mg	1.33 [0.46, 3.81]	1.40 [0.83, 2.34]
ENTRUST: Edoxaban 60mg/30mg	1.34 [0.47, 3.84]	1.39 [0.83, 2.33]
PIONEER: Rivaroxaban 15/10mg	1.25 [0.34, 4.64]	1.40 [0.86, 2.30]
REDUAL: Dabigatran 110mg	1.88 [0.80, 4.40]	1.22 [0.70, 2.11]
REDUAL: Dabigatran 150mg	1.00 [0.35, 2.84]	1.50 [0.89, 2.51]
**total**	1.38 [0.87, 2.20]	

## Supplementary Materials

The supplementary materials are available online at https://www.mdpi.com/2077-0383/9/4/1120/s1.

## Data Sharing

The datasets used or analyzed during the current study are available from the corresponding author on reasonable request.

The lead author affirms that the manuscript is an honest, accurate, and transparent account of the study being reported; that no important aspects of the study have been omitted; and that any discrepancies from the study as planned (and, if relevant, registered) have been explained.

## Abbreviations

Atrial fibrillation	AF
Percutaneous coronary intervention	PCI
Coronary artery disease	CAD
Vitamin K antagonists	VKAs
Non–vitamin K antagonist oral anticoagulants	NOACs
Dual antiplatelet therapy	DAPT
Acute coronary syndrome	ACS
Myocardial infarction	MI
Stent thrombosis	ST
Triple antithrombotic therapy	TAT
Oral anticoagulants	OAC
Randomized controlled trials	RCTs
Single antiplatelet therapy	SAPT
International Society on Thrombosis and Hemostasis	ISTH
Thrombolysis in Myocardial Infarction	TIMI
Major adverse cardiovascular events	MACE
Confidence interval	CI
Dual antithrombotic therapy	DAT
Hazard ratio	HR
