# Supplementary material for: Non-Vitamin K Oral Anticoagulants (NOAC) versus Vitamin K Antagonists (VKA) for Atrial Fibrillation with Elective or Urgent Percutaneous Coronary Intervention: A Meta-Analysis with a Particular Focus on Combination Type
Source: J Clin Med. 2020 Apr 14;9(4):1120. doi: 10.3390/jcm9041120 (PMC7230168; doi:10.3390/jcm9041120)
Supplement: Supplementary file 1 [file jcm-09-01120-s001.pdf]

Non-Vitamin K Oral Anticoagulants (NOAC) Versus Vitamin K Antagonists (VKA) for Atrial Fibrillation With Elective or Urgent Percutaneous Coronary Intervention: A Meta-Analysis with a Particular Focus on Combination Type

Ceren Eyileten [1], Marek Postula [1], Daniel Jakubik [1], Aurel Toma [2], Dagmara Mirowska-Guzel [1], Giuseppe Patti [3], Giulia Renda [4], Jolanta M. Siller-Matula [1,2]

Supplementary Table 1. The definition of major adverse cardiac events (MACE) reported in respective trials.

Trial Name	Major Adverse Cardiac Events
PIONEER-AF PCI	Composite of cardiac death, MI or stroke.
RE-DUAL PCI	Composite of death, MI, stroke, systemic embolism, or unplanned revascularization by PCI/CABG
ENTRUST AF PCI	Composite of death, MI, stroke, systemic embolic events, or definite stent thrombosis
AUGUSTUS	Composite of death or MI, stroke, definite or probable stent thrombosis or urgent revascularization

MI, myocardial infarction**; CABG**, coronary artery bypass grafting; **PCI,** percutaneous coronary intervention; **PIONEER AF**, open-label, randomized , controlled, multicenter study exploring two treatment strategies of rivaroxaban and a dose-adjusted oral vitamin K antagonist treatment strategy in subjects with atrial fibrillation; **RE-DUAL PCI**, randomized evaluation of dual antithrombotic therapy with dabigatran versus triple therapy with warfarin in patients with non-valvular atrial fibrillation undergoing percutaneous coronary intervention; **AUGUSTUS**, an open-label, 2 x 2 factorial, randomized controlled, clinical trial to evaluate the safety of apixaban vs. vitamin K antagonist and aspirin vs. aspirin placebo in patients with atrial fibrillation and acute coronary syndrome or percutaneous coronary intervention; **ENTRUST-AF-PCI**, edoxaban treatment versus vitamin K antagonist in patients with atrial fibrillation undergoing percutaneous coronary intervention.

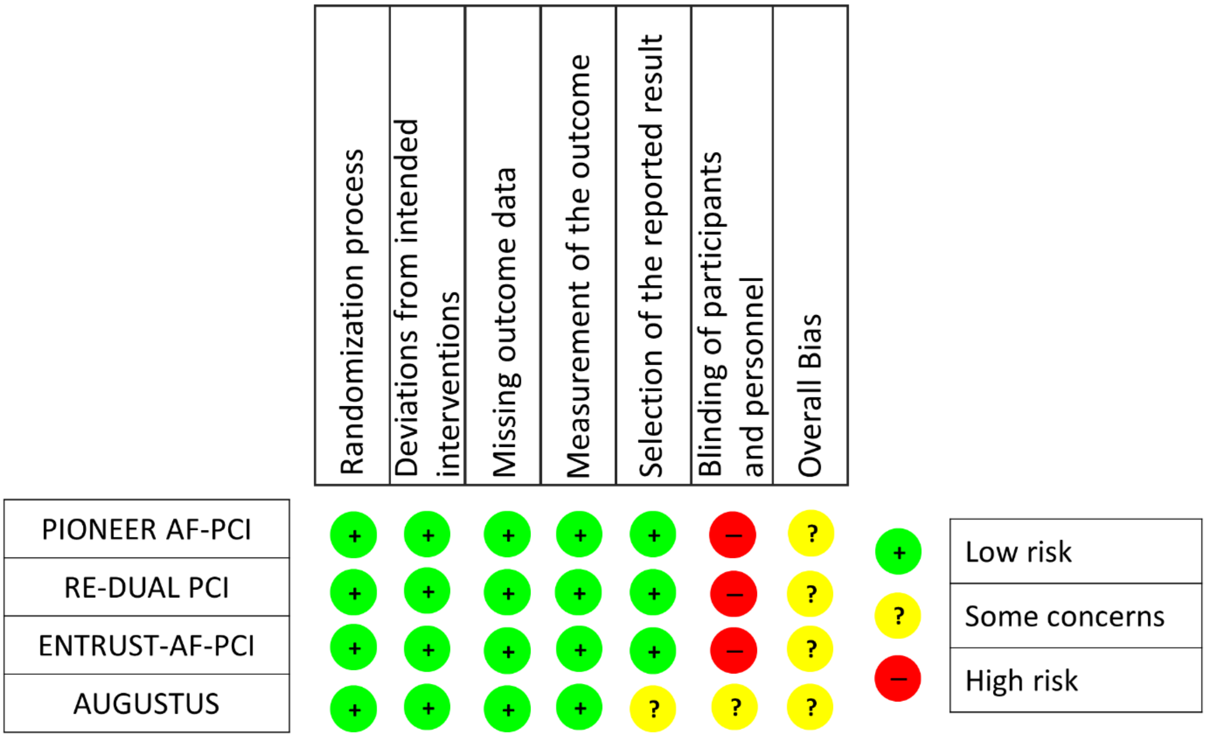

Supplementary Figure 1. Risk of bias summary: risk of bias item for each included RCT according to Cochrane Risk-of-Bias Tool.

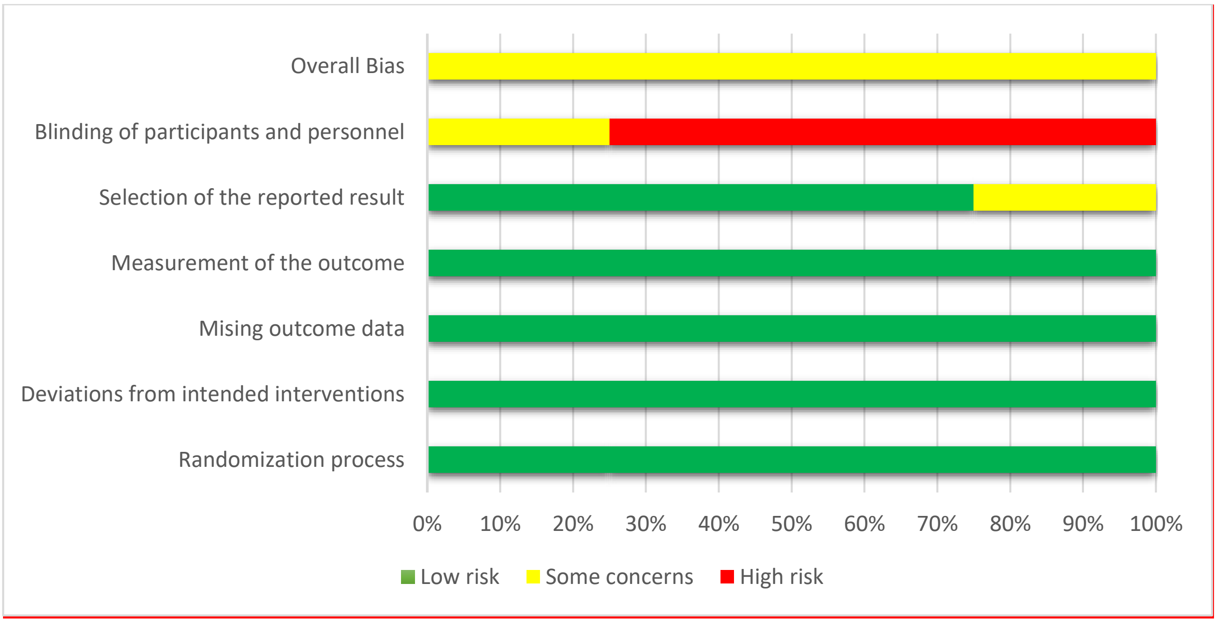

Supplementary Figure 2. Risk of bias graph: each risk of bias item is presented as percentages across all included RCTs.

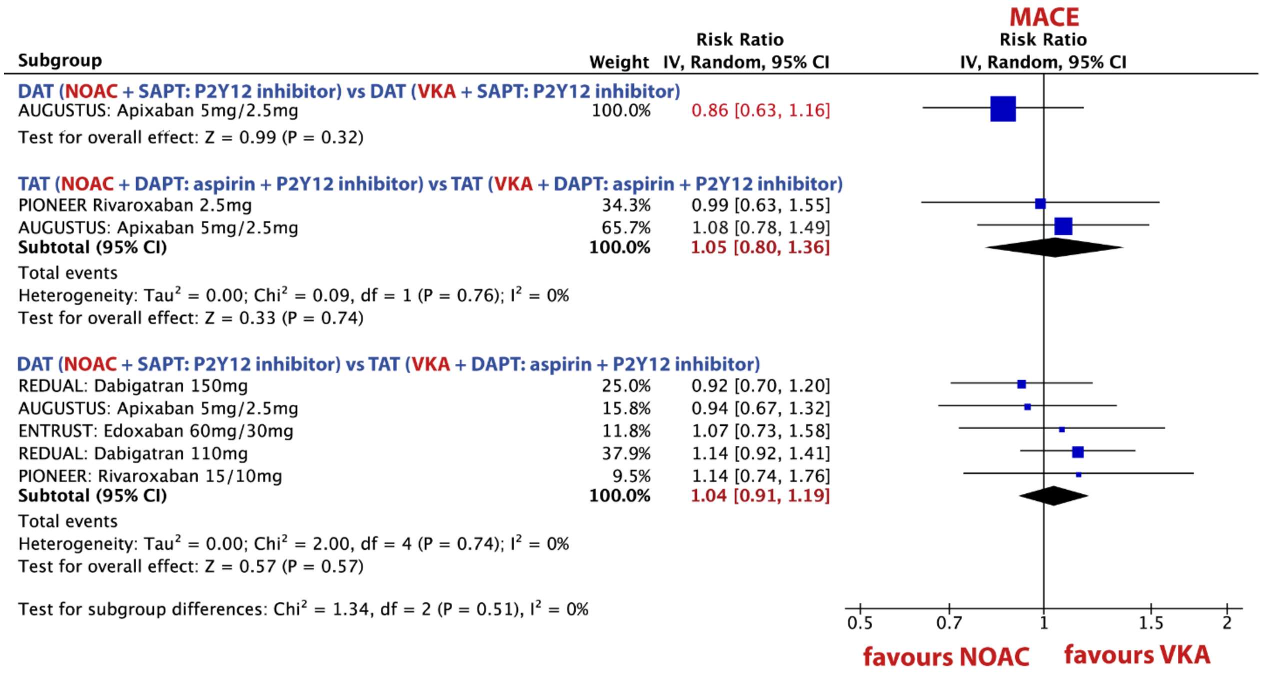

Supplementary Figure 3. Forest plot for the risk of major adverse cardiac events (MACE) in patients treated with non-vitamin K oral anticoagulants~~novel oral anticoagulant~~ (NOAC) vs. vitamin K antagonist (VKA) according to three different combination strategies. Dual antithrombotic therapy (DAT); single antiplatelet therapy (SAPT); triple antithrombotic therapy (TAT); dual antiplatelet therapy (DAPT).

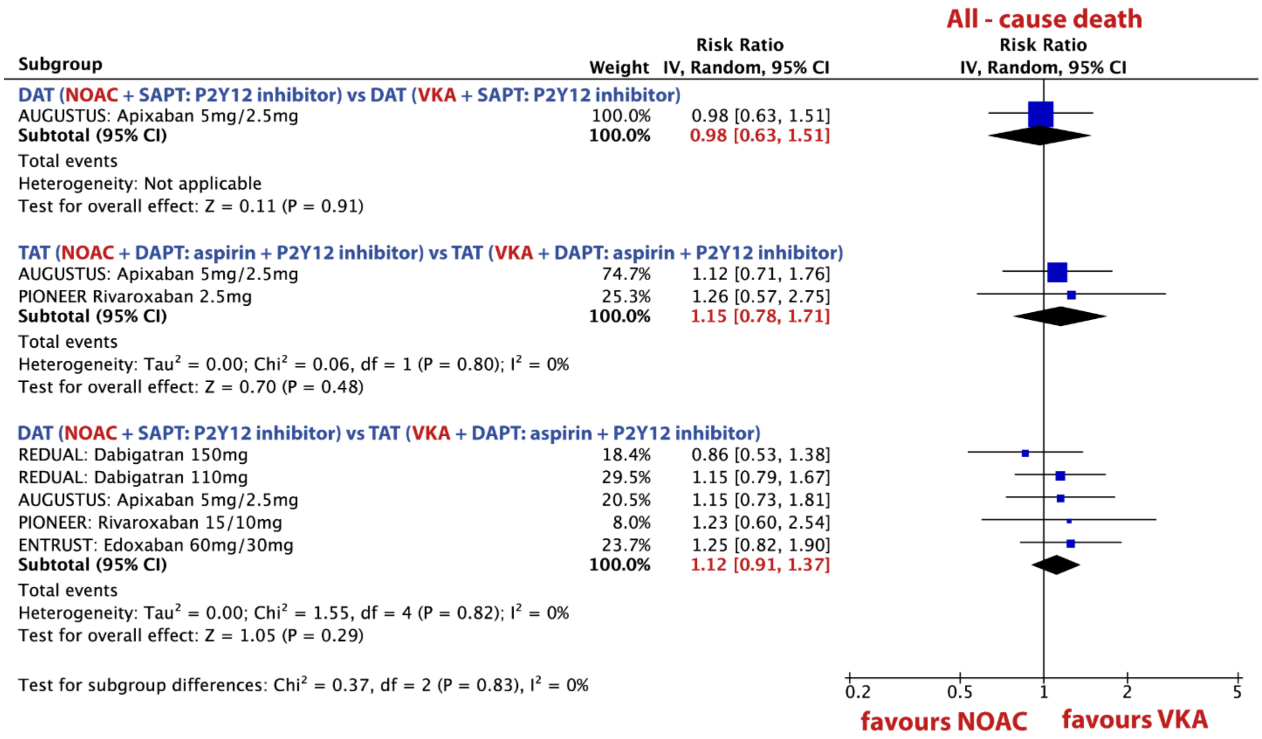

Supplementary Figure 4. Forest plot of all-cause death in patients treated with non-vitamin K oral anticoagulants ~~novel oral anticoagulant~~ (NOAC) vs vitamin K antagonist (VKA) according to three different combination strategies. Dual antithrombotic therapy (DAT); single antiplatelet therapy (SAPT); triple antithrombotic therapy (TAT); dual antiplatelet therapy (DAPT).
